# Capecitabine plus oxaliplatin in the treatment of metastatic colorectal cancer at Tygerberg Hospital: a retrospective study

**DOI:** 10.11604/pamj.2022.42.141.31234

**Published:** 2022-06-22

**Authors:** Solomon Kibudde, Waleed Begg

**Affiliations:** 1 Department of Clinical and Radiation Oncology, Tygerberg Hospital, Stellenbosch University, Cape Town, South Africa,; 2 Department of Radiotherapy, Uganda Cancer Institute, Kampala, Uganda

**Keywords:** Colorectal cancer, capecitabine plus oxaliplatin, chemotherapy

## Abstract

**Introduction:**

a capecitabine and oxaliplatin drug combination regimen has shown a survival benefit in patients with advanced colorectal cancer, yet its administration represents an attractive option for low resource settings. This study aimed to describe the therapeutic utility, efficacy and safety of a capecitabine plus oxaliplatin drug combination in patients with colorectal cancer.

**Methods:**

a review of medical records of sixty adult patients with histological diagnosis of colorectal cancer at Tygerberg Hospital between June 2012 and June 2017 was conducted. The overall response rate was assessed after a three cycle regime of capecitabine and oxaliplatin with the progression-free survival (PSF) results estimated using the Kaplan-Meier methods.

**Results:**

among the 60 participants identified over the study period, the median age was 53 years with 45% being female (n=27). Records showed that 58.33% of patients had the colon as the primary site and 68.33% of patients had synchronous liver metastases at presentation. On average, all patients received 6 cycle regimes of capecitabine and oxaliplatin. Sixty percent of the patients received this treatment regime with palliative intent while in the radical-intent group, equal numbers of patients received the regime as either neoadjuvant or adjuvant. A liver resection was also performed in 20 patients (31.8%). The overall response rate was 69.6% with 13 patients attaining a complete response. Disease progression was reported in 30.4% and the 1-year progression free survival was 44.5% (95% CI: 0.31-0.57) while the 2-year progression free survival was 25.1% (95% CI: 0.14-0.38). Regarding safety, thrombocytopenia was the most frequent adverse event (18.5%) and overall, 15.1% of patients experienced grade 3 and 4 toxicity.

**Conclusion:**

a drug combination of capecitabine and oxaliplatin showed a good overall response rate and survival particularly in patients with resectable colorectal liver metastases.

## Introduction

A capecitabine and oxaliplatin regimen has shown a survival benefit in several phase III trials among patients with advanced colorectal cancer [1]. Capecitabine (Xeloda) is an oral fluoropyrimidine with similar efficacy to bolus 5-fluorouracil used in the first-line treatment of advanced colorectal cancer. It is largely recommended in combination with oxaliplatin, internationally recognized by several treatment guidelines [2]. The combination of capecitabine plus oxaliplatin (XELOX) compared to infusional 5-fluorouracil, leucovorin plus oxaliplatin (FOLFOX) has shown similar response rate, progression-free survival, and overall survival in several phase III clinical trials [1]. The safety of the two regimens differs considerably as 5-fluorouracil, leucovorin plus oxaliplatin (FOLFOX) is associated with higher grade 3 and 4 neutropenia/ granulocytopenia and febrile neutropenia while capecitabine plus oxaliplatin (XELOX) is associated relatively more with grade 3 diarrhoea and grade 3 hand-foot syndrome [3,4]. Also, the administration of capecitabine plus oxaliplatin (XELOX) does not involve continuous infusions and hence it´s an attractive option for limited-resource settings. Lastly, oxaliplatin is effective against fluoropyrimidine resistant forms of colorectal cancer. However, the choice of treatment regimen depends on the physician and patient preferences hence the importance of local data on efficacy and safety. The main objectives of this study were: 1) to describe the therapeutic utility of capecitabine plus oxaliplatin in terms of the treatment intent (palliative or curative) and its treatment duration; 2) to determine the objective response rate of patients to capecitabine plus oxaliplatin; 3) to determine the progression-free survival (PFS) and safety profile of capecitabine plus oxaliplatin regimen.

## Methods

**Study design**: this study was carried out by reviewing the health records of adult patients with a histological diagnosis of colorectal cancer at Tygerberg Hospital. We reviewed hospital records of patients with metastatic colorectal cancer treated with capecitabine and oxaliplatin at our clinic between June 1^st^, 2012, and June 30^th^ 2017.

**Setting**: the study was conducted at the gastrointestinal tract oncology clinic at the department of clinical and radiation oncology, Tygerberg Hospital in Cape Town, South Africa. All patients were discussed in a meeting of a multi-disciplinary team (MDT) that included a hepatobiliary surgeon, clinical oncologist, general surgeons and radiologist. Eligible patients received oxaliplatin at a dose of 130mg/m^2^ as a continuous infusion over 4 hours on the first infusion and subsequent infusions for 2 hours on day 1 of each cycle. Capecitabine was administered orally in two divided doses recommended at 12 hours interval of a daily dose of 2000mg/m^2^ on days 1-14 of every cycle. This was done every 3 weeks for the duration specified as per treatment intent. Before every treatment cycle, patients underwent physical examination with a full blood count, serum chemistries and a targeted assessment for side effects associated with chemotherapy. A computed tomography (CT) scan of the chest, abdomen, and pelvis was performed after the third cycle of chemotherapy to assess response.

**Participants**: the study eligibility criteria were 18 years of age or older with a histological diagnosis of colorectal cancer (colon cancer or rectal cancer), evidence of metastatic disease and treatment with capecitabine plus oxaliplatin-based regimen. Patients who were treated with single-agent capecitabine (Xeloda) and/or patients who received a combination of 5-fluorouracil, leucovorin and oxaliplatin (FOLFOX) regimens were excluded from this study.

**Variables**: the predictable variables were age, sex, performance status, tumour stage, carcinoembryonic antigen (CEA) levels, and sites of metastases, treatment modalities while outcome variables were objective response rate, progression-free survival and safety. Objective response to treatment was defined as the sum of patients attaining complete response (CR), partial response (PR), and/or stable disease (SD). CR is defined as complete disappearance of all disease, PR is defined as ≥ 30% reduction in the diameter of target lesions, SD is defined as a change not meeting criteria for response or progression and PD is defined as a ≥ 20% increase in sum taking as reference the smallest sum on scans or appearance of new lesions. The primary endpoint was progression-free survival (PFS) which we defined as the interval from the start of treatment to the first evidence of progression or death from any cause. Patients surviving without progression were censored at the date of their last hospital visit. Time to progression was defined as the time from the date of the first cycle of chemotherapy to the date of documented evidence of disease progression. Patients were assessed at every visit and adverse events from chemotherapy were documented and graded according to the Common Terminology Criteria for Adverse Events version 3 (CTCAE V3) by the National Cancer Institute (NCI) [5]. Haematology results were reviewed to verify the grade of toxicity reported. If the clinician did not report the grade, we retrospectively evaluated the severity of symptoms reported to assign the toxicity grade.

**Data sources/measurements**: response to treatment was evaluated by comparing pre-treatment CT scan with a post-treatment CT scan using the Response Evaluation Criteria in Solid Tumours (RECIST). This measurement was performed by the radiologist.

**Study size**: no sample size estimation was done due to the retrospective design of this study. Seventy-Five (75) patients were treated with a combination of capecitabine plus oxaliplatin between June 2012 and June 2017 with sixty (60) patients meeting the inclusion criteria.

**Statistical methods**: response rates were reported as proportions and compared using the chi-square test. A p-value of less than 0.05 was regarded as significant. Rates of progression-free survival (PFS) at 12 and 24 months were estimated using the Kaplan-Meier methods and compared by the log-rank test. All the data were entered using Epidata version 3.1 and transferred to Stata version 14 for statistical analysis.

**Ethical considerations**: our study protocol was approved by the Stellenbosch University Health Research Ethics Committee (HREC, Reference #: 1454) and the Research Ethics Committee of Tygerberg Hospital (Reference #: S17/10/193). The study was done following the declaration of Helsinki and Good Clinical Practice guidelines as well as the South African department of health guidelines on ethics in health research.

## Results

**Figure 1:**
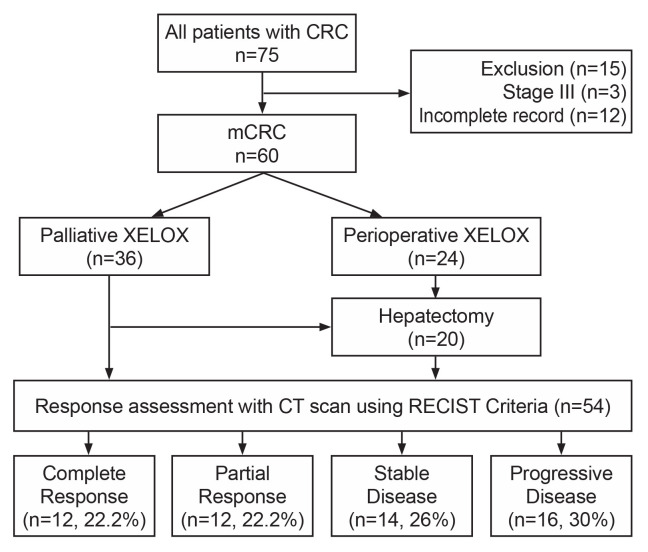
patient flow chart

**Participants**: from June 2012 to June 2017, a total of 75 patients were treated with a combination of oxaliplatin plus capecitabine at Tygerberg Hospital. We excluded 3 patients with stage III disease and 12 patients whose records were not sufficiently accessible for this analysis resulting in only 60 patients who met the inclusion criteria. Thirty-six (36) patients were treated by palliative intent while 24 patients were treated by curative intent (metastatectomy plus neoadjuvant and/or adjuvant XELOX). Liver resection was performed in 20 patients (31.8%) with 18 patients initially treated by curative intent and 2 patients in the palliative intent group (Figure 1). Twenty-seven (45%) were women and the overall median age of the patients was 53 years range (25-77 years). Over half of these patients had the primary tumour site as the colon (58.33%) (Table 1). The majority (69.5%) had synchronous metastases with the liver as the commonest site (66.67%) followed by lungs (15%) and less frequently other sites like the bone, peritoneum, and non-regional nodes. Fifty-nine (93.7%) participants received surgery for the primary tumour, 23 patients (38.3%) received radiotherapy to the primary and all patients received the XELOX regimen as part of treatment. In the 36 patients receiving the XELOX regimen with palliative intent, 75% (27 out of 36 patients) received it as first-line treatment and the rest as second-line treatment. 57 of the 60 (96.61%) participants had a histological diagnosis of adenocarcinoma and the commonest subtype was mucinous (n=12, 20.34%), followed by acinar/tubular and non-mucinous subtypes. Only 3 (5.08%) patients had BRAF testing performed. The majority of patients had locally advanced tumours with T3 tumour (n=38, 63.33%), and T4 tumour (n=11, 18.33%). Twenty-eight patients (46.67%) had N2 nodal disease while 16 patients (26.67%) had N1 nodal status.

**Table 1:** clinical and pathological characteristics

Characteristic	All patients n=60 (%)	Treatment intent		p-value
		Palliative n = 36 (%)	Radical n =24 (%)	
**Age**				0.48
Mean (SD)	52.1 ± 11.7	49.6 ± 12.5	55.9 ± 9.4	
Gender				0.244
Male	33 (55.0)	22 (61.1)	11 (45.8)	
Female	27 (45.0)	14 (38.9)	13 (54.2)	
**ECOG PS**				0.635
≤1	53 (94.6)	31 (93.9)	22 (95.7)	
>1	3 (5.4)	2 (6.1)	1 (4.3)	
**Primary tumor site**				**0.008**
Colon	35 (58.3)	26 (72.2)	9 (37.5)	
Rectal	25 (41.7)	10 (27.8)	15 (62.5)	
**Type of Mets**				0.123
Synchronous	41 (69.5)	27 (77.1)	14 (58.3)	
Metachronous	18 (30.5)	8(22.9)	10(41.7)	
**Tumour location**				0.687
Right	12(35.3)	8(32.0)	4(44.4)	
Left	22(64.7)	17(68.0)	5(55.6)	
**Site of Mets**				**0.001**
Liver only	40(66.7)	18(50.0)	22(91.7)	
Other	20(33.3)	18(50.0)	2(8.3)	
**No. of Mets**				
≤2	32(54.2)	13(36.1)	19(82.6)	**<0.001**
>2	27(45.8)	23(63.9)	4(17.4)	
**Pre-treatment CEA**				0.060
Normal (≤ 5)	29(50.0)	14(40.0)	15(65.2)	
Elevated (>5)	29(50.0)	21(60.0)	8(34.8)	
**Cycles of XELOX**				**0.032**
≤6	44 (73.3)	30 (83.3)	14(58.3)	
>6	16(26.7)	6(16.7)	10(41.7)	

All patients received a combination of oxaliplatin plus capecitabine with only two patients receiving the addition of bevacizumab (Avastin). The average number of cycles of chemotherapy was 6 (IQR 1-9 cycles). In this study, we found the XELOX regimen was used more frequently for palliation (60%), followed by neoadjuvant (18.33%) and adjuvant intent (13.33%). A small proportion of patients (n=5, 8.33%) received neoadjuvant chemotherapy followed by hepatectomy with sequential adjuvant chemotherapy. In this cohort, liver resection was performed in 20 patients (31.8%, 95% CI: 0.21-0.45), with 18 patients initially treated by curative intent and 2 patients in the palliative-intent group. Of the 60 patients, two-thirds of them (n=36, 60%) who received chemotherapy by palliative intent with oxaliplatin plus capecitabine prescribed as the first line in 73.68% patients and second line in 26.32% patients.

### Outcomes

**Figure 2:**
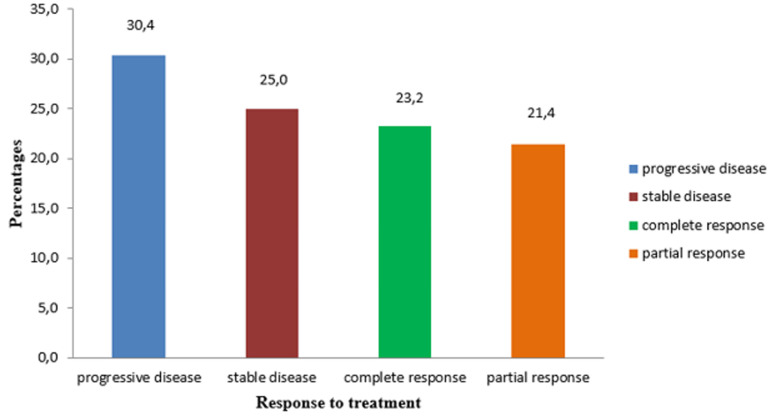
response to treatment with XELOX in patients with mCRC at Tygerberg Hospital

**Treatment response**: the overall response rate (ORR) was 69.6% after 3 cycles of XELOX regimen, representing the sum of patients with either complete response (CR) or partial response (PR) or stable disease (SD) (Figure 2). Thirteen patients attained radiological complete response. Disease progression was observed in 30.4% patients, 95% CI (0.19-0.44) and it was the main reason for discontinuation of chemotherapy. The response was not recorded in 3.1% of patients largely due to loss to follow up.

**Figure 3:**
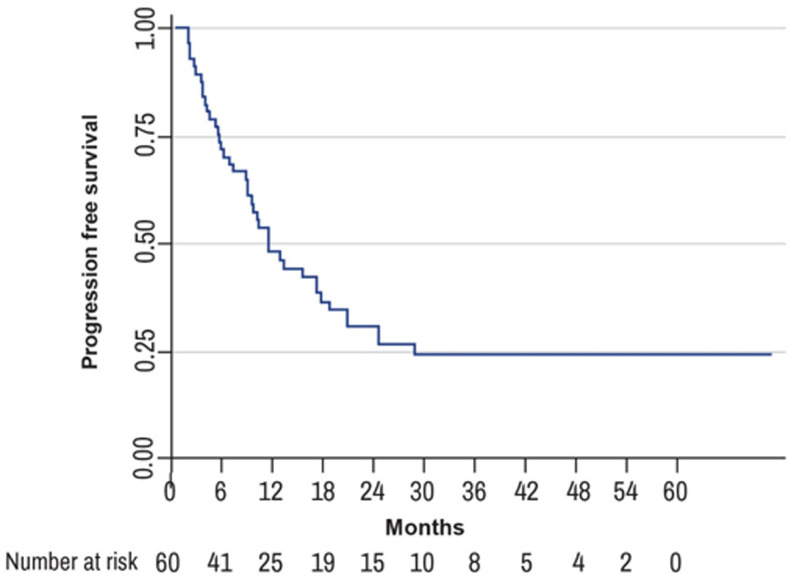
progression-free survival (PFS) at 12 and 24 months

**Figure 4:**
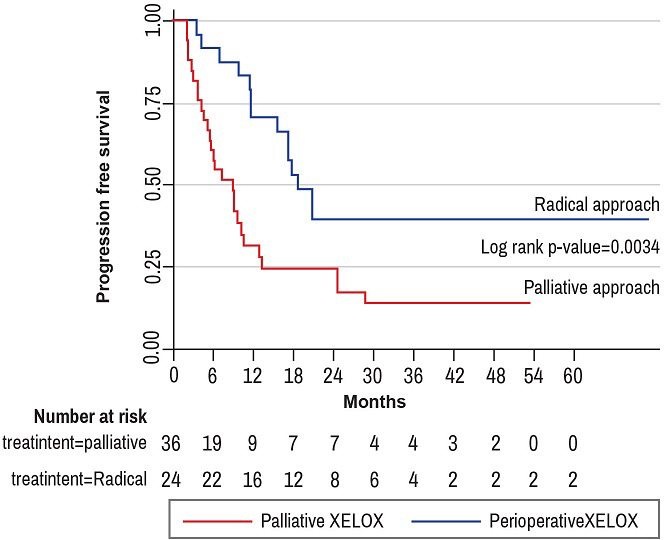
progression-free survival (PFS) by XELOX treatment intent

**Progression-free survival**: overall, 38.7% of study participants experienced disease progression at one year of follow up. The one-year progression-free survival (1-year PFS) was 44.5% (95% CI: 0.31-0.57) while the two year PFS was 25.1% (95% CI: 0.14-0.38). The median time to disease progression was 6 months in the palliative-intent group as compared to 17 months in the radical-intent group (Figure 3). The common sites of disease recurrence were liver (25%), lung (13.6%), bowel (4.6 %), and lymph nodes (2.3%). Death was observed in 25.8% of patients, with more events of death in the palliative-intent group as compared to the radical-intent group. Overall, the median survival time was 11.7 months, 95% CI: 8.93-17.27 [IQR (5.7-24.6)]. In the palliative-intent group, the median survival time was 9 months, 95% CI: 5.23-10.23 [IQR (4.2-13.4)] as compared to a median survival time of 18 months, 95% CI: 11.67-35.90 [IQR (11.7-35.9)] in the radical-intent group with a statistical significance of P-value equal to 0.0034 (Figure 4).

**Predictors of survival**: in this cohort, patients that received XELOX containing chemotherapy by radical intent, and receiving at least 6 cycles of the prescribed treatment, demonstrated statistical significance with p-values 0.002 (HR 2.8, 95% CI [1.5-5.3]) and 0.011 (HR 0.4, 95% CI[0.2 - 0.8]) respectively. Furthermore, surgery (surgical resection of the primary site was associated with an increase in disease-free survival with a p-value 0.021 (HR 0.4, 95% CI 0.2-0.9). Conversely, variables including age, CEA, site of metastases, number of metastases, performance status, and hepatectomy did not attain statistical significance as predictors of progression-free survival in this cohort (Table 2). However, an age greater than 65 years, female gender and liver resection showed clinical significance in predicting survival with HRs of 0.98 (95% CI [0.98-1.00]), 0.65 (95% CI [0.34-1.23]) and 0.43 (95% CI [0.11-1.64]) respectively but did not attain statistical significance.

**Table 2:** univariate and multivariate analysis for predictors of progression-free survival

		Univariate analysis			Multivariate analysis		
Variable	Subgroup	HR	95% CI	p-value	HR	95% CI	p-value
**Age**		0.98	0.98-1.00	0.096			
**Sex**	Male	1					
	Female	0.65	0.34-1.23	0.186			
**CEA**	Normal (≤ 5)	1			1.0		
	Elevated (>5)	1.56	0.83-2.91	0.165	3.05	1.21-7.72	**0.018**
**Primary site**	Colon	1					
	Rectum	1.06	0.57-1.99	0.851			
**Site of Metastases**	Liver only	1					
	Other	1.30	0.68-2.50	0.432			
**No Metastases**	≤2	1					
	>2	1.60	0.87-2.97	0.133			
**PS**	≤1	1					
	>1	0.61	0.06-6.31	0.681			
**Hepatectomy**	No	1					
	Yes	0.43	0.11-1.64	0.219			
**Intent**	Radical	1					
	Palliative	2.55	1.36-4.78	**0.004**			
**Cycles XELOX**		0.81	0.70-0.95	**0.009**	0.60	0.45-0.80	**0.001**
**Metastases**	Synchronous	1					
	Metachronous	0.72	0.39-1.32	0.285			
**Location**	Right	1			1.0		
	Left	1.81	0.80-4.10	0.158	2.46	0.98-6.22	0.056
**Colectomy**	No	1			1.0		
	Yes	0.36	0.17-0.78	**0.009**	11.0	0.997-121.09	0.050
**Radiotherapy**	No	1					
	Yes	1.17	0.61-2.23	0.631			

* 1.0 is the reference category

**Safety, treatment duration and delays**: we observed a total of 126 events with 15.1% of grade 3 and 4 adverse events as well as a discontinuation of treatment due to unacceptable toxicity in 25% of patients. Overall, nearly 65.1% (41 of 63) received at least six cycles of XELOX regimen. The most frequent toxicities were thrombocytopenia occurring in 18.5% of patients followed by nausea and vomiting in 14.3% (Table 3). Hand-foot syndrome was observed in 14.3% of patients and peripheral neuropathy was documented in 14.3% of patients. Overall 15.1% of patients experienced grade 3 and 4 levels of adverse events with the most frequent cause being nausea and vomiting. Chemotherapy was discontinued in 23.3% of patients due to unacceptable toxicity and the leading cause was nausea and vomiting. One patient developed chest pain and was diagnosed with life-threatening arrhythmias leading to discontinuation of oxaliplatin and capecitabine. Of the 30 patients receiving XELOX, 96.7% did not complete the recommended eight (8) courses of chemotherapy. And treatment delays were documented in 24.2% of the study participants. There were 16 events of mortality that occurred during or within thirty (30) days after completion of treatment with XELOX regimen.

**Table 3:** toxicities, grade, and frequency by treatment intent

Treatment-related toxicities	All events, n (%)	Palliative approach, n (%)		Curative approach,n (%)		p-value
		Grade 1 & 2	Grade 3 & 4	Grade 1 & 2	Grade 3 & 4	
**Haematological**						
Neutropenia	17(14.3)	6(85.7)	1(14.3)	9(90.0)	1(10.0)	0.669
Anaemia	9(7.6)	2(66.7)	1(33.3)	6(100)	0(0)	0.333
Thrombocytopenia	22(18.5)	5(83.3)	1(16.7)	13(81.3)	3(18.8)	0.708
**Non-haematological**						
Nausea & vomiting	17(14.3)	7(77.8)	2(22.2)	6(75.0)	2(25.0)	0.665
Diarrhoea	20(16.8)	8(80.0)	2(20.0)	8(80.0)	2(20.0)	0.709
Peripheral neuropathy	16(13.4)	7(100)	0(0)	8(88.9)	1(11.1)	0.562
Hand-foot syndrome	17(14.3)	6(85.7)	1(14.3)	10(100)	0(0)	0.412
Chest pain	1(0.8)	0(0)	1(100)	0(0)	0(0)	

## Discussion

Our findings demonstrate an overall response rate (ORR) of 69.6% after 3 cycles of capecitabine plus oxaliplatin. The progression-free survival at 12 months and 24 months in this cohort was 44.5% and 25.1% respectively. The most striking finding was the difference in survival based on treatment intent which strongly supports a firm correlation between resection of liver metastases and survival. Interestingly, the two-year progression-free survival was 25.1%, a figure that closely corroborates with findings from a nation-wide survey across oncology centres in South Africa in which the 2-year progression-free survival was 30% [6]. Furthermore, it was interesting to find that survival data at 2 years in our cohort was similar to reports from several clinical trials among patients with metastatic colorectal cancer. In the NO16968 study [2], probably the largest randomized trial to compare overall survival (OS) for capecitabine plus oxaliplatin regimen in patients with metastatic CRC was 19.8 months. Our findings confirm reports from previous studies which reported that surgical resection of colorectal cancer liver metastases increases overall survival by over 50% and offers a high probability for a cure [7-10]. In the radical/perioperative XELOX group, the median 2-year progression-free survival was 18 months as compared to 9 months (p-value of 0.0034) among patients that received palliative XELOX. This finding is in close agreement with earlier results by Nordlinger et al.,(2013) who reported a 3-year progression-free survival rate of 25% in patients with resectable colorectal cancer liver metastases that received perioperative chemotherapy with oxaliplatin, folinic acid and 5-fluorouracil (FOLFOX4) [11]. Similarly, the excision of lung metastases translates in 5-year OS rates of 30-50% [12].

Receiving at least six (6) cycles of capecitabine plus oxaliplatin was associated with a progression-free survival benefit of more than 9 months (p-value 0.0218). On average most patients received 6 cycles of XELOX chemotherapy. Ideally, patients with metastatic colorectal cancer can be treated until disease progression or unacceptable toxicity or at least 8 cycles after resection of colorectal cancer liver metastases. This prescription pattern was reported in several oncology centres across South Africa in which most patients received 6 cycles of oxaliplatin containing chemotherapy. We also found the addition of bevacizumab (Avastin) to improve survival as compared to XELOX alone (p-value≤ 0.001). This finding concurs with reports from several clinical trials in which bevacizumab demonstrated improved efficacy in terms of response rates (RR), PFS and OS benefit when combined with 5-FU, capecitabine, irinotecan, or oxaliplatin [13-15]. Two patients in this cohort accessed Avastin® through their medical aid insurance fund. Other factors associated with survival were limited to the female gender. We did not demonstrate an association between survival and previously described variables including young age, site of metastases, number of metastases, or performance status [16]. Among patients with irresectable colorectal cancer liver metastases, the use of capecitabine plus oxaliplatin has been associated with conversion into resectable lesions and eventually improvement in survival. The rate of colorectal cancer metastasectomy was 83.33% among patients treated by radical intent (n=24); however two additional patients initially with unresectable colorectal cancer liver metastases successfully underwent surgery. Notably four patients (16.7%) treated radically never received surgery due to possible disease progression. This proportion is slightly higher than other studies probably because it was outside clinical trials. In the EORTC 40983 trial, 12(7%) patients experienced disease progression during neoadjuvant chemotherapy. Overall the rate of disease progression was 30.4% and this emerged as a frequent contributor to treatment discontinuation.

Presentation with metastases is a poor prognostic factor for patients with colorectal cancer. Other risk factors include the presence of extra hepatic metastases, a higher number of liver metastases, positive portal nodes and uncontrolled primary tumour. In line with existing literature, over half of our patients had liver metastases at presentation [17] probably attributed to tumour spread via the portal system. Secondly, the rate of resectable liver metastases was about 30% which is similar to rates in several trials [9]. The multidisciplinary team discussion is essential for improving clinical care and outcomes particularly in patients with advanced colorectal cancer. Patients that had a colectomy performed had an overall survival benefit as compared to patients that did not receive resection of the primary tumour with a hazard ratio 0.36 (95% CI: 0.17-0.78, p-value = 0.009). This finding is supported by findings from a pooled analysis of four randomized trials of first-line chemotherapy in patients with non-resectable stage IV CRC that confirmed that a history of resection of the primary is independently associated with an overall survival benefit [18]. Overall the proportion of patients receiving capecitabine plus oxaliplatin regimen was low as compared to other regimens like 5-fluorouracil plus leucovorin probably due to local access policy at the time. A similar prescription pattern has been reported in several oncology centres in South Africa where only 32% of patients received XELOX and in 80% of participants, the intent of treatment was palliative [6].

**Safety of XELOX in patients with CRC at Tygerberg Hospital**: the reported incidence of grade 3 and 4 adverse events was relatively low due to several reasons including the retrospective nature of the study that compromised the reporting and grading of common adverse events as well as the attrition rate of patients that did not receive six or more cycles of capecitabine plus oxaliplatin. With regards to the safety of capecitabine plus oxaliplatin regimen in our cohort, the findings rhymed with reports from two large meta-analyses were the incidence of grade 3 and 4 thrombocytopenia, as well as grade 3 and 4 hand-foot syndrome was high in capecitabine plus oxaliplatin group [3, 4]. Neuropathy is a well-documented side effect of oxaliplatin with a rate of 64% in several centres in South Africa. In our study the rate was lower (14.3%) probably due to under-documentation. In the NO16966 study [2], the 60-day all-cause mortality rates were 2.3% (capecitabine plus oxaliplatin). In this cohort, we observed a 30-day all-cause mortality rate high but not directly attributable to XELOX combination chemotherapy.

**Limitations**: our study had some limitations but it provides unique insight into the management of metastatic colorectal cancer in a resource-constrained setting. To begin with, this was a retrospective chart review with limited sample size and hence limited power to draw certain associations. Secondly, it was difficult to ascertain adherence to oral capecitabine since patients received treatment on an outpatient basis. An additional observation was the difficulty to find toxicity data regarding rare or unreported adverse events during the study period. Thirdly, it is plausible that patients that were lost to follow-up probably due to transport issues, socio-economic issues, and other co-morbidities that could have affected compliance and ability to follow-up. Lastly, the optimal timing of the response to treatment was not precise enough. A follow-up scan was booked after 3-4 cycles after which a decision was made to continue or discontinue further treatment with capecitabine plus oxaliplatin. Despite these limitations, our study had several strengths. Although it was a retrospective study, we found reliable data regarding documentation of adverse events associated with capecitabine plus oxaliplatin regarding the grade/severity.

## Conclusion

A drug combination of capecitabine plus oxaliplatin showed a good overall response rate which translated into a good progression-free survival in our cohort. Patients with resectable colorectal cancer liver metastases derived higher benefit from chemotherapy with capecitabine plus oxaliplatin in terms of progression-free survival as compared to the group with irresectable colorectal cancer liver metastases. The administration of at least six cycles of capecitabine plus oxaliplatin was associated with fewer events of mortality or disease progression as compared to receiving less than six cycles of chemotherapy. Although few patients received bevacizumab {Avastin®} in combination with capecitabine plus oxaliplatin which showed a significant survival benefit compared to capecitabine plus oxaliplatin alone. The use of capecitabine plus oxaliplatin resulted in the conversion of irresectable liver metastases to resectable lesions resulting in a significant survival benefit. Liver metastases may have higher chemo sensitivity as compared to metastases in other sites. Overall, the proportion of grade III and IV toxicities associated with capecitabine plus oxaliplatin was relatively low, supporting the safety and tolerability of this regimen. Our experience at Tygerberg shows that capecitabine plus oxaliplatin is safe and effective in patients with metastatic colorectal cancer, and when used in patients with resectable colorectal cancer liver metastases there is a two-fold improvement in survival. In summary, capecitabine plus oxaliplatin is a very active combination for the first-line treatment of colorectal cancer and has a tolerable safety profile in patients with metastatic colorectal cancer at Tygerberg Hospital.

### What is known about this topic

- Approximately 20% of patients with CRC present with synchronous metastases at diagnosis and about 30% of patients treated with curative intent develop metachronous metastases commonly in the liver and lungs;
- In routine clinical practice, the median progression-free survival is estimated at 5.8-7.8 months while the overall survival at 17-20 months.

### What this study adds

- Our findings demonstrate a good overall response rate (ORR) of 69.6% to the capecitabine plus oxaliplatin regimen;
- The progression-free survival was 44.5% and 25.1% at 12-24 months respectively;
- Surgical resection of colorectal cancer liver metastases resulted in a 50% increase in overall survival.
